# Comparative genomic analysis of duplicated homoeologous regions involved in the resistance of *Brassica napus* to stem canker

**DOI:** 10.3389/fpls.2015.00772

**Published:** 2015-09-25

**Authors:** Berline Fopa Fomeju, Cyril Falentin, Gilles Lassalle, Maria J. Manzanares-Dauleux, Régine Delourme

**Affiliations:** ^1^Institut National de la Recherche Agronomique, UMR1349 IGEPPLe Rheu, France; ^2^Agrocampus Ouest, UMR1349 IGEPPLe Rheu, France

**Keywords:** oilseed rape, *Leptosphaeria maculans*, homoeologous QTL, comparative genomics, gene ontology, genome duplications

## Abstract

All crop species are current or ancient polyploids. Following whole genome duplication, structural and functional modifications result in differential gene content or regulation in the duplicated regions, which can play a fundamental role in the diversification of genes underlying complex traits. We have investigated this issue in *Brassica napus*, a species with a highly duplicated genome, with the aim of studying the structural and functional organization of duplicated regions involved in quantitative resistance to stem canker, a disease caused by the fungal pathogen *Leptosphaeria maculans*. Genome-wide association analysis on two oilseed rape panels confirmed that duplicated regions of ancestral blocks E, J, R, U, and W were involved in resistance to stem canker. The structural analysis of the duplicated genomic regions showed a higher gene density on the A genome than on the C genome and a better collinearity between homoeologous regions than paralogous regions, as overall in the whole *B. napus* genome. The three ancestral sub-genomes were involved in the resistance to stem canker and the fractionation profile of the duplicated regions corresponded to what was expected from results on the *B. napus* progenitors. About 60% of the genes identified in these duplicated regions were single-copy genes while less than 5% were retained in all the duplicated copies of a given ancestral block. Genes retained in several copies were mainly involved in response to stress, signaling, or transcription regulation. Genes with resistance-associated markers were mainly retained in more than two copies. These results suggested that some genes underlying quantitative resistance to stem canker might be duplicated genes. Genes with a hydrolase activity that were retained in one copy or R-like genes might also account for resistance in some regions. Further analyses need to be conducted to indicate to what extent duplicated genes contribute to the expression of the resistance phenotype.

## Introduction

Polyploidization is a common mode of evolution in flowering plants which occurs through genome merging (allopolyploidy) or genome doubling (autopolyploidy) and results in an increased gene set (Doyle et al., 2008; Wang et al., 2012). The evolution of duplicated genes after whole-genome duplication (WGD) has been studied extensively. In various plant species, a large proportion of duplicated genes are lost after a polyploidization event, a phenomenon called fractionation or diploidization, while a smaller proportion remains in a duplicated state (Cheng et al., 2012; Jiang et al., 2013). Several hypotheses have been put forward to describe the fate of duplicated genes after WGD, including loss of function, neo-functionalization, sub-functionalization or functional redundancy (Freeling, 2008; Edger and Pires, 2009; Freeling, 2009; Wang et al., 2012).

The loss/retention of duplicated genes is not a random process and, from studies in various plant species, it appears to depend on gene functional category. Thus for example, in *Arabidopsis thaliana*, over-retained genes are involved in basic cellular machinery, nucleotide-sugar metabolism, signal transduction or regulatory functions, while the diploidized genes are involved in DNA repair, tRNA ligation or defense (Blanc and Wolfe, 2004). The loss/retention of duplicated genes might also depend on their parental origin. In this case, one of the parental genomes is more likely to retain genes and has a higher gene density than the other(s) genome(s). This phenomenon, referred to as biased fractionation, has been demonstrated in several species including *Zea mays* (Schnable et al., 2011), *Triticum aestivum* (Pont et al., 2013), *Arabidopsis thaliana* (Thomas et al., 2006), and *Brassica rapa* (Cheng et al., 2012; Tang et al., 2012). Furthermore, expression bias, as described by Wang et al. (2011), has been reported in allopolyploid species: when considering homoeologous gene pairs in allopolyploids, mRNA transcripts from the sub-genome that retains more genes tend to be more highly expressed and thus contribute more to the total transcriptome (Bardil et al., 2011; Schnable et al., 2011; Yoo et al., 2013; Woodhouse et al., 2014).

In several polyploid crop species such as wheat, soybean, cotton and strawberry, it has been shown that duplicated regions were involved in controlling complex agronomical traits (Axelsson et al., 2001; Rong et al., 2007; Liu et al., 2011; Combes et al., 2012; Lerceteau-Kohler et al., 2012). However, there is a lack of studies dissecting the genomic structure and function of these duplicated regions. A recent study in soybean showed that one major QTL (Quantitative Trait Loci) involved in the control of oil and protein seed content on chromosome 20 and its homoeologous region on chromosome 10, were divergent in gene content and gene density (Severin et al., 2011; Lestari et al., 2013). Both expression bias and expression equivalence were observed at the transcriptomic level in these homoeologous regions: out of the 89 homoeologous gene pairs expressed in both regions, 40% showed bias (higher transcript accumulation) toward the homoeologs on chromosome 10. To fully understand both the genetic control and the mechanisms underlying complex traits in polyploid plant species, it is worthwhile to take into account the evolutionary processes through the dissection and comparison of the genomic regions underlying homoeologous duplicated QTL.

*Brassica napus* is an outstanding model for investigating structural and/or functional comparisons of duplicated regions involved in the control of complex traits. Firstly, the *B. napus* genome is highly duplicated. Indeed, *B. napus* (2n = 4x = 38, genome AACC) is an allotetraploid species formed from the hybridization between *Brassica rapa* (2n = 2x = 20, A genome) and *Brassica oleracea* (2n = 2x = 18, C genome) (U, 1935). *Brassica* ancestors have undergone two duplication events (named α and β) and two triplication events of which the most recent is specific to the *Brassica* clade (Jenczewski et al., 2013). These WGD events, along with the merger of the two progenitor genomes, have resulted in a large number of duplicated regions in the *B. napus* genome. Secondly, the organization of duplications in the *B. napus* genome has been well characterized. The ancestral Brassicaceae genome was reconstructed in 24 genomic blocks (A–X) also called the ancestral karyotype blocks (AK blocks). Theses blocks have been identified in *B. rapa* (Schranz et al., 2006; Cheng et al., 2013), *B. oleracea* (Kaczmarek et al., 2009), and *B. napus* (Parkin, 2011; Delourme et al., 2013). Moreover, the structural organization of the hexaploid *Brassica* ancestor genome was determined from whole-genome sequence analyses of the *B. rapa* (Tang and Lyons, 2012; Cheng et al., 2013) and *B. oleracea* (Liu et al., 2014; Parkin et al., 2014) genomes. The ancestral *Brassica* genome contains three sub-genomes showing different levels of fractionation: the LF sub-genome corresponds to the less fractionated genome with the highest gene density, the MF1 sub-genome is the moderately fractionated sub-genome with moderate gene density and sub-genome MF2 is the most fractionated genome with the lowest gene density (Cheng et al., 2013; Liu et al., 2014; Parkin et al., 2014). The organization of these sub-genomes has not yet been elucidated in *B. napus*. However, the recently released genomic sequence of *B. napus* showed good collinearity between the A/C genomic sequences of *B. napus* and the A and C genomes of *B. rapa* and *B. oleracea* (Chalhoub et al., 2014). The organization of LF, MF1 and MF2 can thus be extrapolated to the *B. napus* genome. Thirdly, *B. napus* is closely related to the model plant species *Arabidopsis thaliana* [diverged ~20 million years ago (MYA)] thus allowing functional divergences within *B. napus* duplicated regions to be explored further based on the function of the *A. thaliana* orthologous genes. This relatedness between *A. thaliana* and *B. napus* has been successfully used in previous comparative genetic and genomic analyses to identify candidate genes underlying QTL or to find putative functions of targeted genes (Cai et al., 2012; Zou et al., 2012). In *B. napus*, homoeologous regions were shown to be involved in the control of important traits such as seed glucosinolate content (Howell et al., 2003), flowering time (Wang et al., 2009), yield-related components (Chen et al., 2007) and resistance to sclerotinia stem rot (Zhao et al., 2006; Wei et al., 2014). Comparative genomic analyses of homoeologous *B. napus* regions involved in polygenic traits would then give the opportunity to study the impact of genome duplications on the structural and functional organization of such regions in a highly duplicated genome.

Stem canker caused by the fungus *Leptosphaeria maculans* is one disease of oilseed rape causing serious losses in Europe, Australia and North America (West et al., 2001; Fitt et al., 2006). Both qualitative and quantitative resistances were identified in *B. napus* or in related species (Delourme et al., 2006; Hayward et al., 2012; Raman et al., 2012). Qualitative resistance is based on a gene-for-gene interaction, which is expressed from the seedling stage. More than 10 specific resistance genes (R genes) have been identified in *B. napus* and related Brassica species *B. rapa, B. juncea*, and *B. nigra* (*Rlm1–11, RlmS, LepR1-4*) (Yu et al., 2005, 2008, 2013; Delourme et al., 2006; Van de Wouw et al., 2009; Balesdent et al., 2013), some of which are organized in clusters on *B. napus* chromosomes (Delourme et al., 2004, 2006). Since this resistance is total, it exerts strong selection pressure on the fungal populations that rapidly adapt and varieties whose resistance is based solely on such major genes lose their effectiveness only three-four years after their release (Brun et al., 2000; Li et al., 2003; Rouxel et al., 2003). Quantitative adult-plant resistance, which is a partial, polygenic resistance mediated by QTL is considered as non-race specific (Delourme et al., 2006) and more durable but its effectiveness varies between cropping seasons due to environmental conditions. Qualitative resistance protects the plant from development of leaf lesions at the young plant stage and subsequently prevents the development of stem canker at the adult plant stage. Thus, effective detection of quantitative resistance in field conditions is only possible in the absence of effective R genes. The use of quantitative resistance alone or in combination with qualitative resistance is a way to maximize the effectiveness and durability of the resistance (Brun et al., 2010). These findings highlight the interest and need to strengthen quantitative resistance studies in order to help breeding and improve disease management. Genetic studies of quantitative resistance showed that many QTL in different genetic backgrounds controlled this resistance (Pilet et al., 1998, 2001; Kaur et al., 2009; Jestin et al., 2011, 2012, 2015; Raman et al., 2012). Recently, using linkage and genome-wide association (GWA) analyses, we showed that a large proportion of regions (at least 44%) involved in this quantitative resistance corresponded to duplicated homoeologous regions. These regions were located mainly in duplications of the five AK blocks E, J, R, U, and W (Fopa Fomeju et al., 2014). In the present study, we conducted a comparative genomic analysis of these duplicated homoeologous regions involved in the control of the quantitative resistance to stem canker in *B. napus*. We investigated the similarity/divergence of the duplicated regions in terms of gene content and putative functions of retained genes. We took advantage of the recently released *B. napus* genome sequence (Chalhoub et al., 2014) for the structural analysis of the duplicated regions. *B. napus*—*A. thaliana* relatedness was used to explore gene ontology (GO) categories and the function of genes located in the duplicated regions. According to the functions of the genes that were retained in all duplicated regions or were specific to some regions, we discussed the putative functions associated to the stem canker quantitative resistance.

## Materials and methods

### Association mapping panels, genotyping, and phenotyping data

A panel of 170 winter *B. napus* varieties was used (Table S1). The panel comprises modern oilseed rape (OSR) varieties with low seed erucic acid and glucosinolate content (00 quality) and old varieties with high seed erucic acid and high glucosinolate content (++ quality). The varieties were chosen to represent a wide range of winter oilseed rape diversity and derived from different breeding programs. The absence of any effective specific resistance genes conferring total resistance in our field trial conditions was also checked in order to only evaluate the level of quantitative resistance. This was deduced from cotyledon tests using isolates carrying known *AvrLm* genes (Balesdent et al., pers. comm.). Thus, we excluded OSR varieties known as carrying *Rlm7*, a highly effective gene that was recently introduced in OSR varieties in France. This panel was genotyped with single nucleotide polymorphism (SNP) markers using a *Brassica* 60K SNP BeadChip Array (Liu et al., 2013) and evaluated for resistance to stem canker in a field trial in 2013. One hundred and nine OSR varieties were common to a panel of 116 OSR varieties phenotyped for resistance to stem canker in a field trial in 2006 (Table S1) (Fopa Fomeju et al., 2014).

DNA was isolated from young leaves and DNA extracted using the DNeasy 96 Plant Kit (Qiagen, Courtaboeuf, France). DNA was quantified with the Quant-iT™ PicoGreen® Assay (Invitrogen, Carlsbad, USA), using the Appliskan multiplate reader (Thermo Scientific, Courtaboeuf, France). Concentrations were adjusted to a minimum of 50 ng/μL and were submitted to a provider, where the Infinium® assay was performed following the manufacturer's protocol (Illumina Inc., San Diego, USA). The automatic allele calling for each locus was accomplished using the Genome Studio software (Illumina Inc., San Diego, USA). The clusters were manually edited when necessary. Technical replicates and signal intensities were controlled and only the most reliable calls were retained.

Stem canker was evaluated at one location in a α-design (3 columns and 5 rows) with three replicates (INRA Le Rheu) in France in 2012/13. In each replicate, three control varieties (“Eurol” and “Goeland” as moderately susceptible and “Jet Neuf” as resistant) were replicated in each row × column block in order to check the homogeneity of the trial. Contaminated residues collected from the previous season were scattered throughout the plots 1 month after sowing in order to ensure homogeneous disease infection. Leaf lesions were assessed on each plot at the autumn using a 0–4 scale according to the number of plants with leaf lesions and the number of leaf lesions per plant. Stem canker severity was assessed 2–3 weeks before harvest (mid-June). Forty plants per plot were uprooted and stem canker was assessed on a 0–9 disease index (DI) (Aubertot et al., 2004). DI increases with crown canker severity, starting from zero for healthy plants to nine for completely lodged plants. The 2006 phenotypic data were obtained from a similar experimental design (Jestin et al., 2011).

### Association mapping

Genome wide association (GWA) analyses were performed on two panels (i) panel 1: the sub-panel of 109 OSR varieties phenotyped in 2006 and (ii) panel 2: the whole panel of 170 OSR varieties phenotyped in 2013. A total of 13,973 informative SNPs that were mapped on our “Darmor-*bzh*” × “Yudal” doubled haploid population was selected for association analyses. GWA was carried out as described in Fopa Fomeju et al. (2014) using the GAPIT package (Lipka et al., 2012). Markers with a major allele frequency (MajAF) greater than 0.95 and genotypes or markers with more than 10% of missing genotyping data were eliminated to prevent analyses bias. We used a mixed linear model taking into account the structure within the panel as a fixed effect, and the relatedness between genotypes as a random effect. The calculation of the P matrix, which took into account the panel structure, was based on Principle Component Analysis (PCA) and was performed with EIGENSTRAT software (Price et al., 2006). The calculation of the kinship, or K, matrix was based on an identity by state matrix for all SNP markers and was directly performed by GAPIT (Lipka et al., 2012). The proportion of false positive associations was controlled by a False Discovery Rate (FDR) test at 10%.

### Delineation of the *A. thaliana* and *B. napus* genomic sequences to investigate

The flanking sequences (100–300 bp, median size: 120 bp) of SNPs were used as queries for BLASTn alignments against *A. thaliana* nucleic sequences in TAIR10 (https://www.arabidopsis.org/) with an E-value threshold of 10^−6^. Best gene hits and the corresponding AK block in *A. thaliana* were anchored on the genetic *B. napus* reference map. Thus for each genetic region with resistance-associated markers in *B. napus*, the corresponding *A. thaliana* gene interval was deduced. We took advantage of our present GWA studies and of the one of Fopa Fomeju et al. (2014) to delineate these intervals. They included all the resistance-associated markers identified in all corresponding *B. napus* duplicated regions.

The genomic regions thus delineated were extracted from the *A. thaliana* genome (https://www.arabidopsis.org/) and aligned against the whole *B. napus* genome sequence (Chalhoub et al., 2014) to find the corresponding orthologous duplicated regions in *B. napus*. This reciprocal alignment was conducted to make sure that (i) we investigated all the corresponding duplicated regions in *B. napus* and (ii) we searched for the same ancestral interval on all the duplicates in the *B. napus* genome. The software MumMer v3.0 (Kurtz et al., 2004) was used to conduct a nucleotide versus nucleotide alignment (“NUCmer” command) between each *A. thaliana* region and the *B. napus* genome. The default alignment parameters were used (http://mummer.sourceforge.net/manual/#nucmer).

For a visual overview of the synteny and the collinearity between the identified *B. napus* duplicated regions thus identified, we used the software MAUVE v2.3.1 (Darling et al., 2010).

### Structural analysis of the duplicated regions with resistance-associated markers

We took advantage of the close relatedness between *B. napus* and *A. thaliana* to identify the duplicated genes in the *B. napus* regions. The annotated *B. napus* genes (Chalhoub et al., 2014) were translated and aligned against the bank of *A. thaliana* proteins (https://www.arabidopsis.org/). When two different translated *B. napus* genes showed the best matches to the same *A. thaliana* protein then we considered that the two *B. napus* genes were likely to be homoeologous (if one gene was on the A genome and the other on the C genome) or paralogous (if the two genes were on the same genome).

We compared the duplicated regions in terms of interval length, number of genes and gene density. For each block, we also estimated the level of retention of *A. thaliana* genes in the duplicated regions. The results were examined in relation to the putative organization of the ancestral sub-genomes LF, MF1, and MF2. The organization of these sub-genomes was reported in *B. napus* from findings in *B. rapa* (Cheng et al., 2013) and *B. oleracea* (Liu et al., 2014; Parkin et al., 2014) genomes. We then looked at the distribution of the retained gene copies in the different duplicated regions in *B. napus*. Indeed, for a given block, the *A. thaliana* genes can be present in one of the duplications in *B. napus* or in up to six duplicated regions. Furthermore, in a given *B. napus* region, genes can be present in one copy (single-copy) or in two or more copies (multi-copies).

### *In silico* functional analysis of the duplicated regions with resistance-associated markers

We deduced Gene Ontology (GO) categories from the *A. thaliana* best blast hits in order to functionally characterize and compare the duplicated *B. napus* regions between each other and with *A. thaliana* orthologous regions. The GO terms were obtained from the most recent release of predicted gene locus in the TAIR database (https://www.arabidopsis.org/).

For GO term enrichment analysis, we used the online analysis tool “Singular Enrichment Analysis” (SEA) from AgriGO (http://bioinfo.cau.edu.cn/agriGO/analysis.php). A Fisher test with the Yekutielli multi-test correction method (significance threshold = 0.05) was used. The GO type chosen was the Plant GO slim. For comparison of GO terms of the genes present in 5–6 vs. 1–2 duplicated regions of our studied five blocks, we used a contingency Chi-2 test (α = 0.001).

Finally, nucleotide binding site—leucine rich repeat (NBS-LRR) genes identified in the *B. napus* genome (Chalhoub et al., 2014), genes predicted in the *B. napus* genome as being putative resistance gene analogs in *A. thaliana*, and known major stem canker resistance genes were searched for in our targeted regions. This last category included the major *LepR3/Rlm2* gene which was cloned and identified as a receptor-like protein (Larkan et al., 2013, 2015) and other *Rlm* genes previously located in *B. napus* maps (Delourme et al., 2006). For the latter ones, the previously identified flanking markers were mapped on our SNP map in order to deduce their putative position.

## Results

### Phenotypic analysis of the *B. napus* panel

The results obtained confirmed that we have mainly assessed quantitative resistance in our panels. Indeed, all oilseed rape varieties in the experiment showed typical leaf lesions in the autumn, indicating their lack of effective specific total resistance conferred by major genes that prevent leaf lesion development. As expected, the varieties showed variability in stem canker resistance with DI ranging from 0.3 (resistant) to 8.4 (susceptible). Analysis of variance showed significant (*P* < 0.001) phenotypic variation among lines and replicates. The proportion of total phenotypic variation which resulted from the genotypic variation within the oilseed rape collection was estimated at 0.94. Analysis of variance on the three control varieties did not show a significant genotype x replicate interaction (α = 0.05). The mean disease index of each variety (Table S1) was then used for GWA.

### GWA and identification of duplicated homoeologous regions associated to stem canker resistance

Association analyses were conducted on the two OSR panels with 13,973 informative SNP markers that were well distributed on our reference genetic map with an average density of 1 SNP every 0.15 centimorgans (cM). The average physical distance between two SNPs on the *B. napus* reference sequence was estimated at 60 kb, based on the physical position of the used SNP (Rousseau-Gueutin, pers. comm.). Linkage disequilibrium (LD) evaluated in the two panels extended up to 1.22 cM in the panel of 109 OSR and up to 1.37 cM in the panel of 170 OSR. GWA analyses revealed 686 and 1019 markers significantly (*p*-value < 0.05) associated with resistance in the 109 and 170 OSR panels, phenotyped in 2006 and 2013, respectively (Table S2). A set of 52 associated SNP markers was common to both panels. After false discovery rate (FDR) correction, no significant associated markers were detected in the 109 OSR panel, and one associated marker located on linkage group (LG) A08 in block U was observed in the 170 OSR panel. Out of the 686 and 1019 resistance-associated markers, 505 (73%) and 467 (45%) were clearly anchored on one AK block (Table S3). The remaining markers were located in regions including several nested blocks or were putatively located on two different blocks.

We compared results obtained in the present two GWA analyses with those published by Fopa Fomeju et al. (2014), and we found 28 and 32 common regions containing resistance-associated markers between the three or two analyses, respectively (Table S3). Of these 60 regions, 27 (45%) corresponded to duplications of the AK blocks that were already identified by Fopa Fomeju et al. (2014) i.e., the blocks E (six regions), J (six regions), R (four regions), U (six regions), and W (four regions). In addition in the present study, resistance-associated markers were identified on the six duplications of the block F. In further analyses we focused on the AK blocks E, J, R, U, and W, as these were consistent between our different studies.

### Structural organization of homoeologous duplicated *B. napus* regions

The *A. thaliana* gene intervals corresponding to the duplicated *B. napus* regions that carry resistance associated markers were delineated (Table 1) and their genomic sequence was extracted. The alignment of the sequence of all the studied AK block intervals to the *B. napus* sequence (Figure S1) revealed the expected number (six duplicated regions) and the expected location (according to the genetic map) of the duplications in the *B. napus* genome. The dotplots indicated that there was a good collinearity between *A. thaliana* and *B. napus* nucleotide sequences. Few rearrangements, mainly inversions, were identified between *A. thaliana* and *B. napus* duplicates. One small translocation appeared to be located on U block between chromosomes C3 and C8. Multiple alignments of the duplicated *B. napus* regions indicated that there was globally a better collinearity between homoeologous regions than between paralogous regions (Figure S2).

**Table 1:** **Interval definition of the five AK genomic blocks investigated in this study**.

**AK block**	***A. thaliana* gene interval**
E	At1g65040-At1g80160
J	At2g32530-At2g45060
R	At5g02540-At5g20980
U	At4g17090-At4g40605
W	At5g49620-At5g55630

*The interval definition is based on the location of resistance-associated markers. Each AK interval is defined by two A. thaliana genes*.

For each block, although the size of the duplicated regions was larger on the C genome than on the A genome, the gene density was higher in the A than in the C regions (Table 2). Both on the A and C *B. napus* genomes, the regions showing the highest number of conserved genes with *A. thaliana* corresponded to regions located on the ancestral less fractionated sub-genome (LF sub-genome) (Table 2). The only exception was for the duplication of AK block J on C4, which was not assigned to the same sub-genome in previous studies by Liu et al. (2014) and Parkin et al. (2014). Likewise, for the duplicated regions of blocks E, J, R, and U on the A genome and of block U on the C genome, the regions with an intermediate number and the lowest number of conserved genes with *A. thaliana* corresponded to the MF1 and MF2 sub-genomes, respectively. For the duplicated regions of block W on the A genome and blocks E, J, R, and W on the C genome, the differences between the number of conserved genes with *A. thaliana* was not significant enough to distinguish between the MF1 and MF2 sub-genomes in these regions. Except for the block E, there were more tested SNPs in the regions located on the A than on the C genome. There was no bias in the distribution of tested markers across the three sub-genomes. The proportion of resistance-associated markers identified in the duplicated regions was similar on the A or C genomes, and on the LF, MF1, and MF2 sub-genomes, except for a higher proportion observed on MF2 on A genome.

**Table 2:** **Structural organization of the duplicated regions of AK blocks E, J, R, U, and W in *B. napus* genome**.

**Expected sub-genome according to (Parkin et al., 2014)**	**LF**	**MF1**	**MF2**	**LF**	**MF1**	**MF2**
E Block	**ChrA07**	**ChrA02**	**ChrA07**	**ChrC06**	**ChrC02**	**ChrC06**
Start (in pb)	18912043	6525027	15644284	28782631	12353161	21211096
End (in pb)	24002405	12403698	18772016	37223546	23287093	28440230
Interval length (in pb)	5090362	5878671	3127732	8440915	10933932	7229134
Number of *B. napus* genes	1051	777	548	1324	908	802
Gene density (nb genes/Mb)	206	132	175	157	83	111
Number of *B. napus* genes without an *A. thaliana* gene hit	69	111	27	191	260	170
Number of *B. napus* genes with an *A. thaliana* gene hit	982	666	521	1133	648	632
Number of conserved *B. napus* genes with the orthologous regions in *A. thaliana* genome	798	500	440	849	430	450
Number of SNP tested	147	95	192	169	154	89
Percentage of resistance associated SNP	21.77	15.79	11.46	6.51	6.49	10.11
J Block	**ChrA05**	**ChrA04**	**ChrA03**	**ChrC04**	**BlocJ**	**ChrC03**
Start (in pb)	699676	15016807	6917392	632943	43520171	9229126
End (in pb)	5838732	19149123	10194192	9495166	48900949	14528271
Interval length (in pb)	5139056	4132316	3276800	8862223	5380778	5299145
Number of B. napus genes	951	823	651	1142	856	789
Gene density (nb genes/Mb)	185	199	199	129	159	149
Number of B. napus genes without an A. thaliana gene hit	70	74	62	199	115	117
Number of B. napus genes with an A. thaliana gene hit	881	749	589	943	741	672
Number of conserved B. napus genes with the orthologous regions in A. thaliana genome	694	589	496	649	512	536
Number of SNP tested	172	123	177	135	95	133
Percentage of resistance associated SNP	0.58	0.00	9.60	2.96	5.26	0.00
	1	0	17	4		
R Block	**ChrA10**	**ChrA02**	**ChrA03**	**ChrC09**	**ChrC02**	**ChrC03**
Start (in pb)	11356437	990124	2231348	39930843	2882942	3138662
End (in pb)	13755613	2330220	3716463	44266268	5141051	5148532
Interval length (in pb)	2399176	1340096	1485115	4335425	2258109	2009870
Number of B. napus genes	499	295	340	635	381	417
Gene density (nb genes/Mb)	208	220	229	146	169	207
Number of B. napus genes without an A. thaliana gene hit	17	17	18	101	47	46
Number of B. napus genes with an A. thaliana gene hit	482	278	322	534	334	371
Number of conserved B. napus genes with the orthologous regions in A. thaliana genome	389	252	247	353	254	272
Number of SNP tested	237	27	104	83	34	45
Percentage of resistance associated SNP	0.84	3.70	8.65	0.00	8.82	0.00
U Block	**ChrA01**	**ChrA03**	**ChrA08**	**ChrC01**	**ChrC07**	**ChrC03**
Start (in pb)	342750	21617651	8000633	904490	36754713	50094421
End (in pb)	8931368	28404044	12976485	14682435	44511125	57998913
Interval length (in pb)	8588618	6786393	4975852	13777945	7756412	7904492
Number of *B. napus* genes	1644	1076	753	1925	1353	728
Gene density (nb genes/Mb)	191	159	151	140	174	92
Number of *B. napus* genes without an *A. thaliana* gene hit	121	121	77	271	167	145
Number of *B. napus* genes with an *A. thaliana* gene hit	1523	955	676	1654	1186	583
Number of conserved *B. napus* genes with the orthologous regions in *A. thaliana* genome	1213	751	545	1216	840	436
Number of SNP tested	267	311	125	256	111	198
Percentage of resistance associated SNP	3.00	0.64	51.20	16.02	6.31	3.54
W block	**ChrA10**	**ChrA02**	**ChrA03**	**ChrC09**	**ChrC02**	**ChrC03**
Start (in pb)	3385159	3848607	4036801–11110331	26106711	6797697	6146917
End (in pb)	9960308	6209759	6201708–11257398	39079875	11891907	8380744
Interval length (in pb)	6575149	2361152	1606719–147067	12973164	5094210	2233827
Number of B. napus genes	645	546	467–28	949	480	367
Gene density (nb genes/Mb)	98	231	216–190	73	94	164
Number of B. napus genes without an A. thaliana gene hit	110	62	32– 2	263	103	50
Number of B. napus genes with an A. thaliana gene hit	535	484	259–196	686	377	317
Number of conserved B. napus genes with the orthologous regions in A. thaliana genome	353	258	276	304	268	272
Number of SNP tested	537	131	81	6	28	126
Percentage of resistance associated SNP	0.19	0.00	0.00	0.00	0.00	0.00

The analysis of the conservation of *A. thaliana* orthologous genes in the duplicated *B. napus* regions (Table 3) showed that (i) the *A. thaliana* genes were retained in one to six duplicated regions of a block and (ii) in a given *B. napus* region, one *A. thaliana* orthologous gene was present in 0, 1 or several copies. Thus, duplicated genes can be retained in two ways: one gene copy can be retained on all the duplicated regions of an AK block and/or several copies of the gene can be located in at least one of the duplicated regions of the AK block. For all the studied blocks, most of the *A. thaliana* orthologs (~60%) were located in one or two duplicated regions of a given block. In contrast, only 4% of the genes were present in all the duplicated regions of a given block and 6% on five of the six duplicated regions (Table 3). In most cases, when gene copies were located in two duplicated regions, one copy was on the A genome and the other was on the C genome (data not shown). Overall, for all AK blocks, a large majority of genes (85%) were maintained in one copy per duplicated region. In addition, the proportion of genes in multiple copies is lower for genes that are located in one region compared with genes that are retained in several duplicated regions. For example, 7% of the genes retained in only one duplicate of block J were found in multiple copies within this duplicated region whereas 36% of the genes that were retained on the six duplicates of block J were in multiple copies on at least one of the six duplicated regions.

**Table 3:** **Distribution of genes in the studied duplicated regions in *B. napus* genome**.

		**E block**		**J block**		**R block**		**U block**		**W block**	
		**Number**	**Percentage**	**Number**	**Percentage**	**Number**	**Percentage**	**Number**	**Percentage**	**Number**	**Percentage**
Retained in 6 regions	Total genes	34	2	50	4	34	5	61	3	25	2
	Single copy	29	85	32	64	20	59	42	69	17	68
	Multi-copies	5	15	18	36	14	41	19	31	8	32
Retained in 5 regions	Total genes	79	5	76	5	34	5	127	6	35	3
	Single copy	56	71	57	75	28	82	99	78	25	71
	Multi-copies	23	29	19	25	6	18	28	22	10	29
Retained in 4 regions	Total genes	261	18	277	20	148	21	391	19	126	10
	Single copy	211	81	217	78	124	84	311	80	87	69
	Multi-copies	50	19	70	25	24	16	80	20	39	31
Retained in 3 regions	Total genes	243	17	229	16	131	19	331	16	151	12
	Single copy	195	80	180	79	103	79	261	79	123	81
	Multi-copies	48	20	49	21	28	21	70	21	28	19
Retained in 2 regions	Total genes	599	42	540	39	235	34	761	36	353	29
	Single copy	519	87	452	84	207	88	631	83	308	87
	Multi-copies	80	13	88	16	28	12	130	17	45	13
Retained in 1 region	Total genes	462	32	505	36	230	33	817	39	520	43
	Single copy	427	92	463	92	213	93	757	93	472	91
	Multi-copies	35	8	42	8	17	7	60	7	48	9
Total	Total genes	1678	100	1677	100	812	100	2488	100	1210	100
	Single copy	1437	86	1401	84	695	86	2101	84	1032	85
	Multi-copies	241	14	286	17	117	14	387	16	178	15

*For each block, the number of orthologous A. thaliana genes has been identified in the duplicated regions (total genes). These genes can be retained in one of the duplicated regions of each block or up to the six duplicated regions. In each region, one gene can be represented in one copy (single copy) or in two or more copies (multi-copies)*.

### Gene ontology term analyses

**Figure 1:**
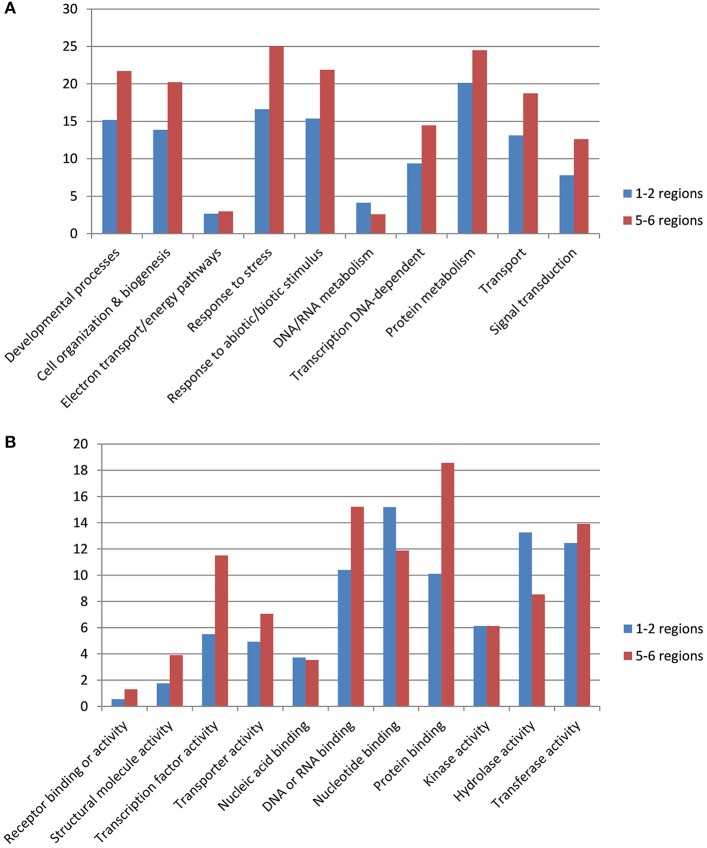
**Gene ontology terms associated to genes retained in 1–2 or 5–6 duplicated regions**. Gene ontology terms for biological process **(A)** and molecular activity **(B)**.

We investigated gene categories in the duplicated regions involved in resistance to stem canker. *B. napus* genes with an ontology deduced from *A. thaliana* orthologs were used to perform GO slim enrichment analyses. No significant enrichment was identified when we compared GO terms of genes located in the duplicated *B. napus* regions with the GO terms of genes located in the corresponding *A. thaliana* orthologous regions. We then compared GO terms of genes located on five and six duplicated regions versus those located on one or two regions in *B. napus*. Among the genes located in five or six duplicated regions (Table S4), there were significantly more genes involved in “developmental processes,” “cell organization & biogenesis,” “response to stress,” “response to abiotic or biotic stimulus,” “transcription DNA-dependent,” “transport,” and “signal transduction” compared with genes located in one duplicated region (Figure 1). Furthermore, among the genes retained in five or six duplicated regions, more genes had molecular functions corresponding to “transcription factor activity,” “protein binding,” and “transferase activity.” In contrast, there were more genes with a molecular function corresponding to “hydrolase activity” within genes located on one or two duplicated regions (Figure 1).

### Structural and functional characterization of genes with resistance-associated markers

**Figure 2:**
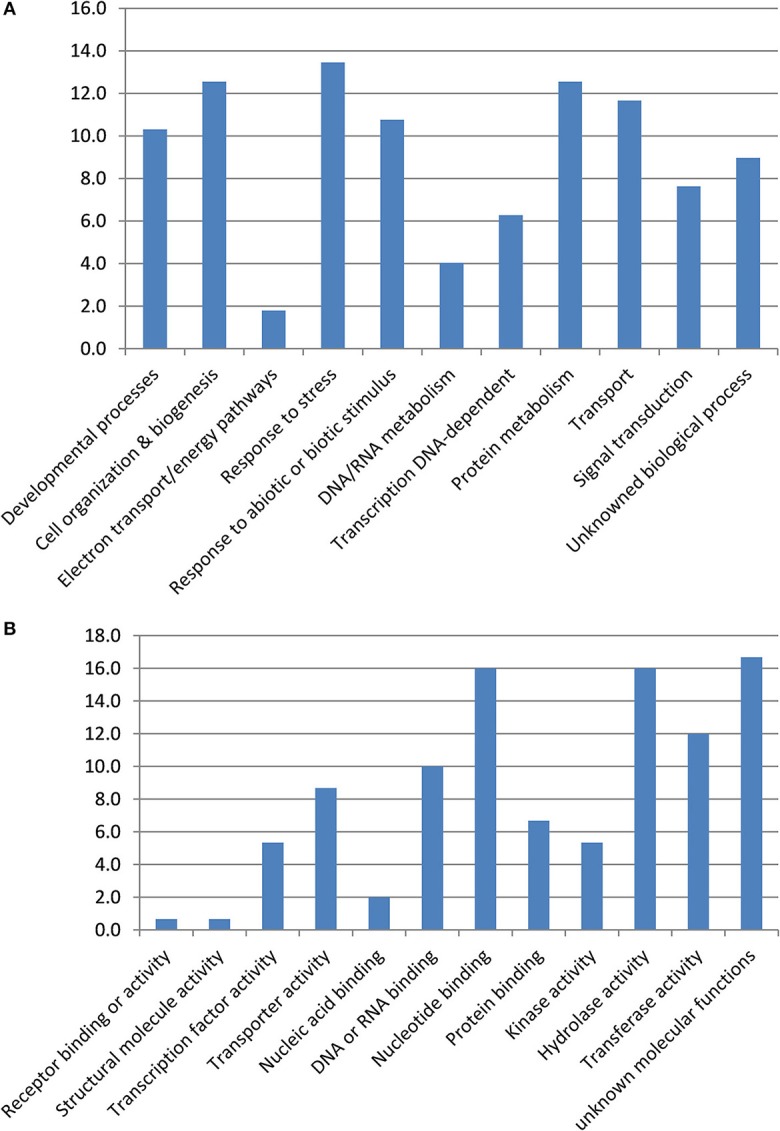
**Gene ontology terms associated to SNP/genes associated to resistance to stem canker**. Gene ontology terms for biological process **(A)** and molecular activity **(B)**.

From the present GWA studies and the one of Fopa Fomeju et al. (2014), we identified 294 resistance-associated markers in the duplicated regions corresponding to the five studied AK blocks. Of these, 148 markers were anchored in *B. napus* genes. They corresponded to 129 genes, of which 124 had a blast hit with *A. thaliana* and their putative function was thus investigated (Table S5). These 124 *B. napus* genes represented a large proportion of the resistance associated markers on the blocks W (65%) and E (51%) and about 30% of resistance-associated markers on blocks J, R, and U. Seven resistance-associated genes were retained in only one duplicated region and the 117 others were retained in two or more duplicated regions. Among these 117 genes, 18 (15%) were present in multiple copies in at least one of the duplicated regions. The 124 resistance-associated genes were mainly related to the biological processes “response to stress,” “cell organization & biogenesis,” and “protein metabolism” (Figure 2). The three main molecular functions found were “hydrolase activity,” “nucleotide binding,” and “transferase activity.” These GO categories described genes located on five or six duplicated regions, except for “hydrolase activity” which was preferentially identified for genes located in one duplicated region.

### Investigation of R-like genes

Out of the 425 NBS-LRR genes identified in the *B. napus* genome (Chalhoub et al., 2014), 135 genes were located in our studied *B. napus* duplicated regions. In addition, we found that 89 *B. napus* genes had a significant hit to NBS-LRR genes or resistance gene analogs (RGA) in *A. thaliana*. These were mostly distributed on duplicated regions corresponding to the blocks E, R, and U. Out of the 224 resistance-like genes (NBS-LRR and RGA), 21 were tested in the association study and four were significantly associated with resistance to stem canker (Table S2). Two were located on block E on LG A7 (LF sub-genome) and C2 and the two others on block U on LG C7. According to genetic linkage maps, the *Rlm3-Rlm4-Rlm7-Rlm9* cluster might be in the neighborhood of block E-LF sub-genome on chromosome A7 and *Rlm1* was assigned to the studied region of block E-MF2 sub-genome on chromosome A7. The approximate mapping position of *Rlm1* spread in a region of 1300 kb according to bridge SNP between linkage map and genomic sequence (Table S2). In this region, 22 resistance-associated markers have been tested and 12 were significantly associated to resistance to stem canker. *LepR3/Rlm2* sequence aligned to the *B. napus* gene GSBRNA2G00135758001 which was located outside our investigated region on the block R on chromosome A10 and was not tested in association mapping.

## Discussion

In this study, we analyzed the structural and functional organization of duplicated regions involved in quantitative resistance of *B. napus* to stem canker. We took advantage of the recently released *B. napus* genome sequence (Chalhoub et al., 2014) for structural analyses and of the *B. napus*—*A. thaliana* relatedness for exploring GO categories and the putative function of genes located in the duplicated regions.

### Duplicated regions of at least five AK blocks are involved in quantitative resistance to stem canker

Our present GWA analyses confirmed that duplicated regions of AK blocks E, J, R, U, and W are involved in quantitative resistance to stem canker as shown in our previous GWA study (Fopa Fomeju et al., 2014). Results from FDR corrected data indicated that there might be false positives among the resistance-associated markers. However, the duplicated regions of the blocks E, J, R, U, and W were identified with three datasets, suggesting that these regions are likely to be “true” quantitative resistance regions. Moreover, at least 15 of these duplicated resistance associated regions (on LG A01, A02, A03, A05, A08, C01, C02, C03, C04, C06, and C07) were located within or in the vicinity of QTL that were previously identified in biparental segregating populations and in a connected multiparental population (Pilet et al., 2001; Jestin et al., 2012, 2015). New potential duplicated regions were highlighted (such as duplications of block F) indicating that, as suggested in (Fopa Fomeju et al., 2014), more than 44% of duplicated genomic regions are involved in the control of this complex trait. This proportion is quite high compared to that observed in other polyploid plant species such as strawberry (Lerceteau-Kohler et al., 2012) or cotton (Rong et al., 2007) for other traits. This could be tightly linked to either the good structural conservation between the *B. napus* A and C genomes (Cheung et al., 2009; Chalhoub et al., 2014), the highly duplicated nature of the Brassicaceae genomes resulting from several WGD events, or the fact that loci involved in defense responses are often over-retained in plants after WGD (Blanc and Wolfe, 2004; Moghe et al., 2014).

### Structural organization of the homoeologous duplicated regions involved in stem canker resistance

All the expected duplicated regions of our targeted blocks were found on the *B. napus* genomic sequence. This reflects the good coverage of the *B. napus* sequence, which was estimated at 79% (Chalhoub et al., 2014). Alignment of *B. napus* duplicated regions revealed better collinearity between homoeologous regions than between paralogous regions, homoeologous duplicated regions having more genes in common than paralogous duplicated regions, as generally observed in the genomes of *B. napus* and its progenitors (Cheung et al., 2009). The size of the extracted duplicated regions varied depending on their location on either the A or C genome. For a given block, the studied regions on the C genome were larger than their homoeologous regions on the A genome. It has been shown that the C genome is larger due to insertions of transposable elements; almost 40% of the C vs. 26% of the A genome corresponds to transposable elements in *B. napus* (Chalhoub et al., 2014). Similar results were observed for the A and C genomes of *B. rapa* and *B. oleracea*, respectively (Cheung et al., 2009; Cheng et al., 2012; Liu et al., 2014).

By analyzing gene distribution in our studied regions, we observed that the *B. napus* duplicated regions with the highest number of retained genes compared with *A. thaliana* were orthologous to regions located on the less fractionated sub-genome (LF) of *B. rapa* (Cheng et al., 2012) and *B. oleracea* (Liu et al., 2014; Parkin et al., 2014). This agrees with the hypothesis that the fractionation of the sub-genomes took place before the *B. rapa*—*B. oleracea* divergence (Tang et al., 2012) and suggests that, probably due to the recentness of *B. napus*, no significant new fractionation levels were observed in this allotetraploid. However, the occurrence of homoeologous exchanges, which may shape fractionation levels by modifying gene number, on the sub-genomes have been reported (Chalhoub et al., 2014), suggesting that fractionation might be ongoing in the *B. napus* genome. The proportions of resistance associated markers on the LF, MF1 and MF2 subgenomes were similar, which suggests that all the subgenomes were involved in the control of the resistance trait. This remains to be further studied since the number of associated markers identified does not provide information on the number of underlying causative genes, nor on their contribution to the phenotype expression.

When considering gene distribution across the duplicated regions, we found that a large majority of genes (60%) were only present in one or two duplicated regions of the AK blocks and that these were mostly present in a single copy per region (on average, 85% of the genes). In general, genes present in two duplicated regions had one copy on the A genome and the other copy on the C genome. In a recent study, De Smet et al. (2013) estimated that 66% of the *B. rapa* genes were single-copy genes. This is consistent with our findings suggesting that these single-copy genes have not resulted from the allotetraploidization event but rather from the long-term evolution of diploid ancestors.

### Functional analysis of the genes located in the duplicated regions involved in resistance to stem canker

Genes located on five or six of the duplicated regions of a given block represented about 10% of the total genes identified. The GO terms of these duplicated genes were related to response to stress, signaling and transcription regulation as identified in other plant species (Blanc and Wolfe, 2004; Paterson et al., 2006). Highly duplicated genes, i.e., genes present in several copies in a given region, were preferentially associated to transport, signal transduction and kinase activity functions. Similar results were reported in other plant species (De Smet et al., 2013; Han et al., 2014). Actually, genes involved in adaptive traits (such as resistance or tolerance to biotic and abiotic stresses) are preferentially over-retained after a WGD event allowing faster evolution and conferring an adaptive advantage to polyploid plants (Pont et al., 2013; Murat et al., 2014; Wu et al., 2014). This can lead to copy number or sequence variation between accessions in the homoeologous and paralogous regions and to phenotype differentiation as shown for flowering-related genes (Schiessl et al., 2014). Twenty-two of the genes retained in five or six duplicated regions were associated to resistance and were related to response to stress and transferase activity. However, at this point, we could not conclude on the involvement of duplicated genes in stem canker resistance due to the fact that only one of the duplicated copies was tested for 77% of the duplicated genes.

Genes maintained in only one duplicated region were enriched in “hydrolase activity” functions. An analysis conducted by Han et al. (2014) in 29 angiosperm genomes indicated that genes in single copy have binding and catalytic activities (including hydrolase activity). Previously, Schranz and Mitchell-Olds (2006) highlighted that genes involved in hydrolase activity were retained as single-copy genes after the alpha duplication in the Brassicaceae lineage. All these results suggest that the bias in retention of genes in single copy is common across different species. Thus our results may reflect a global pattern across the whole genome and may not be specific to the studied regions. Twenty-four associated markers were located in genes present in only one duplicated region and had GO terms related to “hydrolase activity.” Thus, we cannot exclude that genes encoding proteins with hydrolase activity are involved in response to stem canker in some regions. Indeed, the involvement of hydrolase activity has been highlighted in the response to tobacco virus infection (Guo et al., 1998). The HE-1 hydrolase protein may play a role in protection from oxidative damage associated with defense responses. It may also play a role in generating signals for activation of certain defense responses. The relationship between hydrolase activity and plant response to pathogens has been also demonstrated in *A. thaliana* (Kang et al., 2008) and potato (Reddy et al., 2000).

R and R-like genes could also account for resistance in some regions. Indeed, four resistance-associated markers located in NBS-LRR or RGA genes were found on blocks E and U. In addition, the major gene *Rlm1* conferring blackleg resistance in *B. napus* was located in the neighborhood of the resistance-associated markers on the block E, chromosome A7, sub-genome MF2. The effect of this region on resistance could be due either to a residual effect of *Rlm1* gene or to linked genes, as previously suggested for *Rlm2* gene that was shown to co-localize with a resistance QTL to stem canker (Pilet et al., 2001).

## Conclusion and future prospects

We identified a set of genes that were retained in duplicated regions and associated with quantitative resistance to stem canker and many were involved in stress responses, signaling and transcriptional regulation. It was shown in various other species that such genes were over-retained after WGD, indicating a common evolutionary pattern across species. We also found that almost 60% of the genes located in the studied duplicated regions were single- or two-copy genes, as expected in the whole *B. napus* genome, and that some of them e.g., genes with a hydrolase activity were associated with resistance. Thus, we cannot exclude that the genes underlying quantitative resistance to stem canker are not duplicated genes. Results also suggested that R-like genes might account for *B. napus* quantitative resistance to stem canker in some genomic regions. In future studies, the *B. napus* genes that are present in our studied regions but had no hit with *A. thaliana* genes, should be investigated to complete the results. The three *B. napus* sub-genomes (LF, MF1, and MF2) were found to contribute to quantitative stem canker resistance. In wheat, it was suggested that the most fractionated genome (B sub-genome) i.e., the sub-genome which is more sensitive to genome fractionation and more plastic, controls adaptive traits such as reaction to biotic and abiotic stresses (Pont et al., 2013). This has to be further analyzed in oilseed rape by investigating whether all duplicated copies are expressed and equally contribute to phenotype expression and if they have undergone sub-functionalization, which appears to be a major force in maintaining genes in a duplicated state.

## Author contributions

BF performed GWA studies, structural and functional sequence analyses. CF carried out genetic mapping and BLAST analyses for the construction of the integrated map used in this study and the positioning of duplicated blocks on this map. GL contributed to the structural and BLAST analyses. RD and MM helped analyse the results and coordinated the project. BF, RD, and MM wrote the manuscript.

### Conflict of interest statement

The authors declare that the research was conducted in the absence of any commercial or financial relationships that could be construed as a potential conflict of interest.

## References

[B1] Axelsson T. Shavorskaya O. Lagercrantz U. (2001). Multiple flowering time QTLs within several Brassica species could be the result of duplicated copies of one ancestral gene. Genome 44, 856–864. 10.1139/gen-44-5-856 11681610

[B2] Aubertot J. N. Schott J. J. Penaud A. Brun H. Doré T. (2004). Methods for sampling and assessment in relation to the spatial pattern of phoma stem canker (*Leptosphaeria maculans*) in oilseed rape. Eur. J. Plant Pathol. 110, 183–192. 10.1023/B:EJPP.0000015359.61910.3b11681610

[B3] Balesdent M. H. Fudal I. Ollivier B. Bally P. Grandaubert J. Eber F.. (2013). The dispensable chromosome of *Leptosphaeria maculans* shelters an effector gene conferring avirulence towards *Brassica rapa*. New Phytol. 198, 887–898. 10.1111/nph.12178 23406519

[B4] Bardil A. de Almeida J. D. Combes M. C. Lashermes P. Bertrand B. (2011). Genomic expression dominance in the natural allopolyploid *Coffea arabica* is massively affected by growth temperature. New Phytol. 192, 760–774. 10.1111/j.1469-8137.2011.03833.x 21797880

[B5] Blanc G. Wolfe K. H. (2004). Functional divergence of duplicated genes formed by polyploidy during Arabidopsis evolution. Plant Cell 16, 1679–1691. 10.1105/tpc.021410 15208398 PMC514153

[B6] Brun H. Chèvre A. M. Fitt B. D. L. Powers S. Besnard A. L. Ermel M.. (2010). Quantitative resistance increases the durability of qualitative resistance to Leptosphaeria maculans in *Brassica napus*. New Phytol. 185, 285–299. 10.1111/j.1469-8137.2009.03049.x 19814776

[B7] Brun H. Levivier S. Somda I. Ruer D. Renard M. Chèvre A. M. (2000). A field method for evaluating the potential durability of new resistance sources: application to the *Leptosphaeria maculans/Brassica napus* pathosystem. Phytopathology 90, 961–966. 10.1094/PHYTO.2000.90.9.961 18944519

[B8] Cai G. Yang Q. Yang Q. Zhao Z. Chen H. Wu J.. (2012). Identification of candidate genes of QTLs for seed weight in *Brassica napus* through comparative mapping among *Arabidopsis* and *Brassica* species. BMC Genet. 13:105. 10.1186/1471-2156-13-105 23216693 PMC3575274

[B9] Chalhoub B. Denoeud F. Liu S. Parkin I. A. P. Tang H. Wang X.. (2014). Plant genetics. Early allopolyploid evolution in the post-Neolithic *Brassica napus* oilseed genome. Science 345, 950–953. 10.1126/science.1253435 25146293

[B10] Chen W. Zhang Y. Liu X. Chen B. Tu J. Tingdong F. (2007). Detection of QTL for six yield-related traits in oilseed rape (*Brassica napus*) using DH and immortalized F-2 populations. Theor. Appl. Genet. 115, 849–858. 10.1007/s00122-007-0613-2 17665168

[B11] Cheng F. Mandáková T. Wu J. Xie Q. Lysak M. A. Wang X. (2013). Deciphering the diploid ancestral genome of the mesohexaploid *Brassica rapa*. Plant Cell 25, 1541–1554. 10.1105/tpc.113.110486 23653472 PMC3694691

[B12] Cheng F. Wu J. Fang L. Sun S. Liu B. Lin K.. (2012). Biased gene fractionation and dominant gene expression among the subgenomes of *Brassica rapa*. PLoS ONE 7:e36442. 10.1371/journal.pone.0036442 22567157 PMC3342247

[B13] Cheung F. Trick M. Drou N. Lim Y. P. Park J. Y. Kwon S. J.. (2009). Comparative analysis between homoeologous genome segments of *Brassica napus* and its progenitor species reveals extensive sequence-level divergence. Plant Cell 21, 1912–1928. 10.1105/tpc.108.060376 19602626 PMC2729604

[B14] Combes M. C. Cenci A. Baraille H. Bertrand B. Lashermes P. (2012). Homeologous gene expression in response to growing temperature in a recent allopolyploid (*Coffea arabica* L.). J. Hered. 103, 36–46. 10.1093/jhered/esr120 22039298

[B15] Darling A. E. Mau B. Perna N. T. (2010). progressiveMauve: multiple genome alignment with gene gain, loss and rearrangement. PLoS ONE 5:e11147. 10.1371/journal.pone.0011147 20593022 PMC2892488

[B16] De Smet R. Adams K. L. Vandepoele K. Van Montagu M. C. E. Maere S. Van de Peer Y. (2013). Convergent gene loss following gene and genome duplications creates single-copy families in flowering plants. Proc. Natl. Acad. Sci. U.S.A. 110, 2898–2903. 10.1073/pnas.1300127110 23382190 PMC3581894

[B17] Delourme R. Pilet-Nayel M. L. Archipiano M. Horvais R. Tanguy X. Rouxel T.. (2004). A cluster of major specific resistance genes to *Leptosphaeria maculans* in *Brassica napus*. Phytopathology 94, 578–583. 10.1094/PHYTO.2004.94.6.578 18943482

[B18] Delourme R. Chèvre A. M. Brun H. Rouxel T. Balesdent M. H. Dias J. S. (2006). Major gene and polygenic resistance to *Leptosphaeria maculans* in oilseed rape (*Brassica napus*). Eur. J. Plant Pathol. 114, 41–52. 10.1007/s10658-005-2108-9

[B19] Delourme R. Falentin C. Fopa Fomeju B. F. Boillot M. Lassalle G. André I.. (2013). High-density SNP-based genetic map development and linkage disequilibroum assessment in *Brassica napus* L. BMC Genomics 14:120. 10.1186/1471-2164-14-120 23432809 PMC3600037

[B20] Doyle J. J. Flagel L. E. Paterson A. H. Rapp R. A. Soltis D. E. Soltis P. S.. (2008). Evolutionary genetics of genome merger and doubling in plants. Ann. Rev. Genet. 42, 443–461. 10.1146/annurev.genet.42.110807.091524 18983261

[B21] Edger P. P. Pires J. C. (2009). Gene and genome duplications: the impact of dosage-sensitivity on the fate of nuclear genes. Chromos. Res. 17, 699–717. 10.1007/s10577-009-9055-9 19802709

[B22] Fitt B. D. L. Brun H. Barnetti M. J. Rimmer S. R. (2006). World-wide importance of phoma stem canker (*Leptosphaeria maculans* and *L. biglobosa*) on oilseed rape (*Brassica napus*). Eur. J. Plant Pathol. 192, 763–774. 10.1007/s10658-005-2233-5

[B23] Fopa Fomeju B. Falentin C. Lassalle G. Manzanares-Dauleux M. J. Delourme R. (2014). Homoeologous duplicated regions are involved in quantitative resistance of *Brassica napus* to stem canker. BMC Genomics 15:498. 10.1186/1471-2164-15-498 24948032 PMC4082613

[B24] Freeling M. (2008). The evolutionary position of subfunctionalization, downgraded. Genome Dynam. 4, 25–40. 10.1159/000126004 18756075

[B25] Freeling M. (2009). Bias in plant gene content following different sorts of duplication: tandem, whole-genome, segmental, or by transposition. Ann. Rev. Plant Biol. 60, 433–453. 10.1146/annurev.arplant.043008.092122 19575588

[B26] Guo A. Durner J. Klessig D. F. (1998). Characterization of a tobacco epoxide hydrolase gene induced during the resistance response to TMV. Plant J. 15, 647–656. 9778847 10.1046/j.1365-313x.1998.00241.x

[B27] Han F. Peng Y. Xu L. Xiao P. (2014). Identification, characterization, and utilization of single copy genes in 29 angiosperm genomes. BMC Genomics 15:504. 10.1186/1471-2164-15-504 24950957 PMC4092219

[B28] Hayward A. McLanders J. Campbell E. Edwards D. Batley J. (2012). Genomic advances will herald new insights into the Brassica: *Leptosphaeria maculans* pathosystem. Plant Biol. 14, 1–10. 10.1111/j.1438-8677.2011.00481.x 21973193

[B29] Howell P. M. Sharpe A. G. Lydiate D. J. (2003). Homoeologous loci control the accumulation of seed glucosinolates in oilseed rape (*Brassica napus*). Genome 46, 454–460. 10.1139/g03-028 12834062

[B30] Jenczewski E. Chèvre A. Alix K. (2013). Chromosomal and gene expression changes in Brassica Allopolyploids, in Polyploid and Hybrid Genomics, eds Chen J. Birchle J. (Iowa: John Wiley and Sons, Inc.), 171–186

[B31] Jestin C. Lodé M. Domin C. Falentin C. Horvais R. Coedel S. (2011). Association mapping of quantitative resistance for *Leptosphaeria maculans* in oilseed rape (*Brassica napus* L.). Mol. Breed. 27, 190–201. 10.1007/s11032-010-9429-x

[B32] Jestin C. Vallée P. Domin C. Manzanares-Dauleux M. J. Delourme R. (2012). Assessment of a new strategy for selective phenotyping applied to complex traits in *Brassica napus*. Open J. Genet., 2, 190–201. 10.4236/ojgen.2012.24025

[B33] Jestin C. Bardol N. Lodé M. Duffé P. Domin C. Vallée P. (2015). Connected populations for detecting quantitative resistance factors to Phoma stem canker in oilseed rape (*Brassica napus* L.). Mol. Breed. 35, 167 10.1007/s11032-015-0356-8

[B34] Jiang W. K. Liu Y. L. Xia E. H. Gao L. (2013). Prevalent role of gene features in determining evolutionary fates of whole-genome duplication duplicated genes in flowering plants. Plant Physiol. 161, 1844–1861. 10.1104/pp.112.200147 23396833 PMC3613460

[B35] Kaczmarek M. Koczyk G. Ziolkowski P. A. Babula-Skowronska D. Sadowski J. (2009). Comparative analysis of the *Brassica oleracea* genetic map and the *Arabidopsis thaliana* genome. Genome 52, 620–633. 10.1139/G09-035 19767893

[B36] Kang L. Wang Y. S. Uppalapati S. R. Wang K. Tang Y. Vadapalli V.. (2008). Overexpression of a fatty acid amide hydrolase compromises innate immunity in *Arabidopsis*. Plant J. 56, 336–349. 10.1111/j.1365-313X.2008.03603.x 18643971

[B37] Kaur S. Cogan N. Ye G. Baillie R. Hand M. Ling A.. (2009). Genetic map construction and QTL mapping of resistance to blackleg (*Leptosphaeria maculans*) disease in Australian canola (*Brassica napus* L.) cultivars. Theor. Appl. Genet. 120, 71–83. 10.1007/s00122-009-1160-9 19821065

[B38] Kurtz S. Phillippy A. Delcher A. L. Smoot M. Shumway M. Antonescu C.. (2004). Versatile and open software for comparing large genomes. Genome Biol. 5:R12. 10.1186/gb-2004-5-2-r12 14759262 PMC395750

[B39] Larkan N. J. Lydiate D. J. Parkin I. A. Nelson M. N. Epp D. J. Cowling W. A.. (2013). The *Brassica napus* blackleg resistance gene *LepR3* encodes a receptor-like protein triggered by the *Leptosphaeria maculans* effector AVRLM1. New Phytol. 197, 595–605. 10.1111/nph.12043 23206118

[B40] Larkan N. J. Ma L. Borhan M. H. (2015). The *Brassica napus* receptor-like protein RLM2 is encoded by a second allele of the *LepR3*/*Rlm2* blackleg resistance locus. Plant Biotech. J. 13, 983–992. 10.1111/pbi.12341 25644479

[B41] Lerceteau-Köhler E. Moing A. Guérin G. Renaud C. Petit A. Rothan C.. (2012). Genetic dissection of fruit quality traits in the octoploid cultivated strawberry highlights the role of homoeo-QTL in their control. Theor.Appl. Genet. 124, 1059–1077. 10.1007/s00122-011-1769-3 22215248 PMC3304055

[B42] Lestari P. Van K. Lee J. Kang Y. J. Lee S.-H. (2013). Gene divergence of homeologous regions associated with a major seed protein content QTL in soybean. Front. Plant Sci. 4:176. 10.3389/fpls.2013.00176 23761803 PMC3672674

[B43] Li H. Sivasithamparam K. Barbetti M. J. (2003). Breakdown of a *Brassica rapa* subsp. *sylvestris* single dominant blackleg resistance gene in *Brassica napus* rapeseed by *Leptosphaeria maculans* field isolates in Australia. Plant Dis. 87:752 10.1094/PDIS.2003.87.6.752A 30812879

[B44] Lipka A. E. Tian F. Wang Q. Peiffer J. Li M. Bradbury P. J.. (2012). GAPIT: genome association and prediction integrated tool. Bioinformatics 28, 2397–2399. 10.1093/bioinformatics/bts444 22796960

[B45] Liu W. Kim M. Y. Kang Y. J. Van K. Lee Y. H. Srinives P.. (2011). QTL identification of flowering time at three different latitudes reveals homeologous genomic regions that control flowering in soybean. Theor. Appl. Genet. 123, 545–553. 10.1007/s00122-011-1606-8 21660531

[B46] Liu S. Y. Liu Y. M. Yang X. H. Tong C. B. Edwards D. Parkin I. A. P.. (2014). The *Brassica oleracea* genome reveals the asymmetrical evolution of polyploid genomes. Nature Comm. 5, 3930. 10.1038/ncomms4930 24852848 PMC4279128

[B47] Liu L. Qu C. Wittkop B. Yi B. Xiao Y. He Y.. (2013). A high-density SNP map for accurate mapping of seed fibre QTL in *Brassica napus* L. PLoS ONE 8:e83052. 10.1371/journal.pone.0083052 24386142 PMC3873396

[B48] Moghe G. D. Hufnagel D. E. Tang H. Xiao Y. Dworkin I. Town C. D.. (2014). Consequences of whole-genome triplication as revealed by comparative genomic analyses of the wild radish *Raphanus raphanistrum* and three other brassicaceae species. Plant Cell 26, 1925–1937. 10.1105/tpc.114.124297 24876251 PMC4079359

[B49] Murat F. Zhang R. Guizard S. Flores R. Armero A. Pont C.. (2014). Shared subgenome dominance following polyploidization explains grass genome evolutionary plasticity from a seven protochromosome ancestor with 16K protogenes. Genome Biol. Evol. 6, 12–33. 10.1093/gbe/evt200 24317974 PMC3914691

[B50] Parkin I. (2011). Ghosts: comparative mapping in the brassicaceae, in Plant Genetics and Genomics: Crops and Models, Vol. 9, eds Schmidt R. Bancroft I. (New York, NY; Dordrecht; Heidelberg; London: Springer), 153–170.

[B51] Parkin I. A. Koh C. Tang H. Robinson S. J. Kagale S. Clarke W. E.. (2014). Transcriptome and methylome profiling reveals relics of genome dominance in the mesopolyploid *Brassica oleracea*. Genome Biol. 15:R77. 10.1186/gb-2014-15-6-r77 24916971 PMC4097860

[B52] Paterson A. H. Chapman B. A. Kissinger J. C. Bowers J. E. Feltus F. A. Estill J. C. (2006). Many gene and domain families have convergent fates following independent whole-genome duplication events in *Arabidopsis, Oryza, Saccharomyces* and *Tetraodon*. Trends Genet. 22, 597–602. 10.1016/j.tig.2006.09.003 16979781

[B53] Pilet M. L. Delourme R. Foisset N. Renard M. (1998). Identification of loci contributing to quantitative field resistance to blackleg disease, causal agent *Leptosphaeria maculans* (Desm.) Ces. et de Not., in Winter rapeseed (*Brassica* napus L.). Theor. Appl. Genet. 96, 23–30.

[B54] Pilet M. L. Duplan G. Archipiano H. Barret P. Baron C. Horvais R. (2001). Stability of QTL for field resistance to blackleg across two genetic backgrounds in oilseed rape. Crop Sci. 41, 197–205. 10.2135/cropsci2001.411197x

[B55] Pont C. Murat F. Guizard S. Flores R. Foucrier S. Bidet Y.. (2013). Wheat syntenome unveils new evidences of contrasted evolutionary plasticity between paleo- and neoduplicated subgenomes. Plant J. 76, 1030–1044. 10.1111/tpj.12366 24164652

[B56] Price A. L. Patterson N. J. Plenge R. M. Weinblatt M. E. Shadick N. A. Reich D. (2006). Principal components analysis corrects for stratification in genome-wide association studies. Nat. Genet. 38, 904–909. 10.1038/ng1847 16862161

[B57] Raman R. Taylor B. Marcroft S. Stiller J. Eckermann P. Coombes N.. (2012). Molecular mapping of qualitative and quantitative loci for resistance to Leptosphaeria maculans causing blackleg disease in canola (*Brassica napus* L.). Theor. Appl. Genet. 125, 405–418. 10.1007/s00122-012-1842-6 22454144

[B58] Reddy P. S. Kumar T. C. Reddy M. N. Sarada C. Reddanna P. (2000). Differential formation of octadecadienoic acid and octadecatrienoic acid products in control and injured/infected potato tubers. Biochem. Biophys. Acta Mol. Cell Biol. Lipids 1483, 294–300. 10.1016/s1388-1981(99)00191-2 10634945

[B59] Rong J. Feltus F. A. Waghmare V. N. Pierce G. J. Chee P. W. Draye X.. (2007). Meta-analysis of polyploid cotton QTL shows unequal contributions of subgenomes to a complex network of genes and gene clusters implicated in lint fiber development. Genetics 176, 2577–2588. 10.1534/genetics.107.074518 17565937 PMC1950656

[B60] Rouxel T. Willner E. Coudard L. Balesdent M. H. (2003). Screening and identification of resistance to *Leptosphaeria maculans* (stem canker) in *Brassica napus* accessions. Euphytica 133, 219–231. 10.1023/A:1025597622490

[B61] Schiessl S. Samans B. Hüttel B. Reinhard R. Snowdon R. J. (2014). Capturing sequence variation among flowering-time regulatory gene homologs in the allopolyploid crop species *Brassica napus*. Front. Plant Sci. 5:404. 10.3389/fpls.2014.00404 25202314 PMC4142343

[B62] Schnable J. C. Springer N. M. Freeling M. (2011). Differentiation of the maize subgenomes by genome dominance and both ancient and ongoing gene loss. Proc. Natl. Acad. Sci. U.S.A. 108, 4069–4074. 10.1073/pnas.1101368108 21368132 PMC3053962

[B63] Schranz M. E. Lysak M. A. Mitchell-Olds T. (2006). The ABC's of comparative genomics in the Brassicaceae: building blocks of crucifer genomes. Trends Plant Sci. 11, 535–542. 10.1016/j.tplants.2006.09.002 17029932

[B64] Schranz M. E. Mitchell-Olds T. (2006). Independent ancient polyploidy events in the sister families Brassicaceae and Cleomaceae. Plant Cell 18, 1152–1165. 10.1105/tpc.106.041111 16617098 PMC1456871

[B65] Severin A. J. Cannon S. B. Graham M. M. Grant D. Shoemaker R. C. (2011). Changes in twelve homoeologous genomic regions in soybean following three rounds of polyploidy. Plant Cell 23, 3129–3136. 10.1105/tpc.111.089573 21917551 PMC3203428

[B66] Tang H. Lyons E. (2012). Unleashing the genome of *Brassica rapa*. Front. Plant Sci. 3:172. 10.3389/fpls.2012.00172 22866056 PMC3408644

[B67] Tang H. Woodhouse M. R. Cheng F. Schnable J. C. Pedersen B. S. Conant G.. (2012). Altered patterns of fractionation and exon deletions in *Brassica rapa* support a two-step model of paleohexaploidy. Genetics 190, 1563–1574. 10.1534/genetics.111.137349 22308264 PMC3316664

[B68] Thomas B. C. Pedersen B. Freeling M. (2006). Following tetraploidy in an *Arabidopsis* ancestor, genes were removed preferentially from one homeolog leaving clusters enriched in dose-sensitive genes. Genome Res. 16, 934–946. 10.1101/gr.4708406 16760422 PMC1484460

[B69] U N. (1935). Genome analysis in Brassica with special reference to the experimental formation of *B. napus* and peculiar mode of fertilization. Jpn. J. Bot. 7, 389–452.

[B70] Van de Wouw A. P. Marcroft S. J. Barbetti M. J. Li H. U. Salisbury P. A. Gout L. (2009). Dual control of avirulence in *Leptosphaeria maculans* towards a *Brassica napus* cultivar with ‘sylvestris-derived’ resistance suggests involvement of two resistance genes. Plant Pathol. 58, 305–313. 10.1111/j.1365-3059.2008.01982.x

[B71] Wang J. Long Y. Wu B. D. Liu J. Jiang C. C. Shi L.. (2009). The evolution of *Brassica napus* FLOWERING LOCUST paralogues in the context of inverted chromosomal duplication blocks. BMC Evol. Biol. 9:271. 10.1186/1471-2148-9-271 19939256 PMC2794288

[B72] Wang X. Wang H. Wang J. Sun R. Wu J. Liu S.. (2011). The genome of the mesopolyploid crop species *Brassica rapa*. Nat. Genet. 43, 1035–U1157. 10.1038/ng.919 21873998

[B73] Wang Y. Wang X. Paterson A. H. (2012). Genome and gene duplications and gene expression divergence: a view from plants. Year Evol. Biol. 1256, 1–14. 10.1111/j.1749-6632.2011.06384.x 22257007

[B74] Wei D. Mei J. Fu Y. Disi J. O. Li J. Wei Q. (2014). Quantitative trait loci analyses for resistance to Sclerotinia sclerotiorum and flowering time in *Brassica napus*. Mol. Breed. 34, 1797–1804. 10.1007/s11032-014-0139-7

[B75] West J. S. Kharbanda P. D. Barbetti M. J. Fitt B. D. L. (2001). Epidemiology and management of *Leptosphaeria maculans* (phoma stem canker) on oilseed rape in Australia, Canada and Europe. Plant Pathol. 50, 10–27. 10.1046/j.1365-3059.2001.00546.x

[B76] Woodhouse M. R. Cheng F. Pires J. C. Lisch D. Freeling M. Wang X. (2014). Origin, inheritance, and gene regulatory consequences of genome dominance in polyploids (vol 111, pg 5283, 2014). Proc. Natl. Acad. Sci. U.S.A. 111, 6527–6527. 10.1073/pnas.1405833111 PMC3986174 24706847

[B77] Wu P. Shao Z. Wu X.Z. Wang Q. Wang B. Chen J. Q.. (2014). Loss/retention and evolution of NBS-encoding genes upon whole genome triplication of *Brassica rapa*. Gene 540, 54–61. 10.1016/j.gene.2014.01.082 24576745

[B78] Yoo M. J. Szadkowski E. Wendel J. F. (2013). Homoeolog expression bias and expression level dominance in allopolyploid cotton. Heredity 110, 171–180. 10.1038/hdy.2012.94 23169565 PMC3554454

[B79] Yu F. Lydiate D. J. Rimmer S. R. (2005). Identification of two novel genes for blackleg resistance in *Brassica napus*. Theor. Appl. Genet. 110, 969–979. 10.1007/s00122-004-1919-y 15798929

[B80] Yu F. Lydiate D. J. Rimmer S. R. (2008). Identification and mapping of a third blackleg resistance locus in *Brassica napus* derived from *B. rapa* subsp. sylvestris. Genome 51, 64–72. 10.1139/g07-103 18356940

[B81] Yu F. Gugel R. K. Kutcher H. R. Peng G. Rimmer S. R. (2013). Identification and mapping of a novel blackleg resistance locus *LepR4* in the progenies from *Brassica napus* x *B. rapa* subsp. sylvestris. Theor. Appl. Genet. 126, 307–315. 10.1007/s00122-012-1919-2 22733446

[B82] Zhao J. W. Udall J. A. Quijada P. A. Grau C. R. Meng J. L. Osborn T. C. (2006). Quantitative trait loci for resistance to *Sclerotinia sclerotiorum* and its association with a homeologous non-reciprocal transposition in *Brassica napus* L. Theor. Appl. Genet. 112, 509–516. 10.1007/s00122-005-0154-5 16333614

[B83] Zou X. X. Suppanz I. Raman H. Hou J. N. Wang J. Long Y.. (2012). Comparative Analysis of FLC Homologues in Brassicaceae Provides Insight into Their Role in the Evolution of Oilseed Rape. PLoS ONE 7:45751. 10.1371/journal.pone.0045751 23029223 PMC3459951

