# Supplementary material for: Comparative genomic analysis of duplicated homoeologous regions involved in the resistance of Brassica napus to stem canker
Source: Front Plant Sci. 2015 Sep 25;6:772. doi: 10.3389/fpls.2015.00772 (PMC4585320; doi:10.3389/fpls.2015.00772)
Supplement: Supplementary file 6 [file Presentation1.PPTX]

## Slide 1
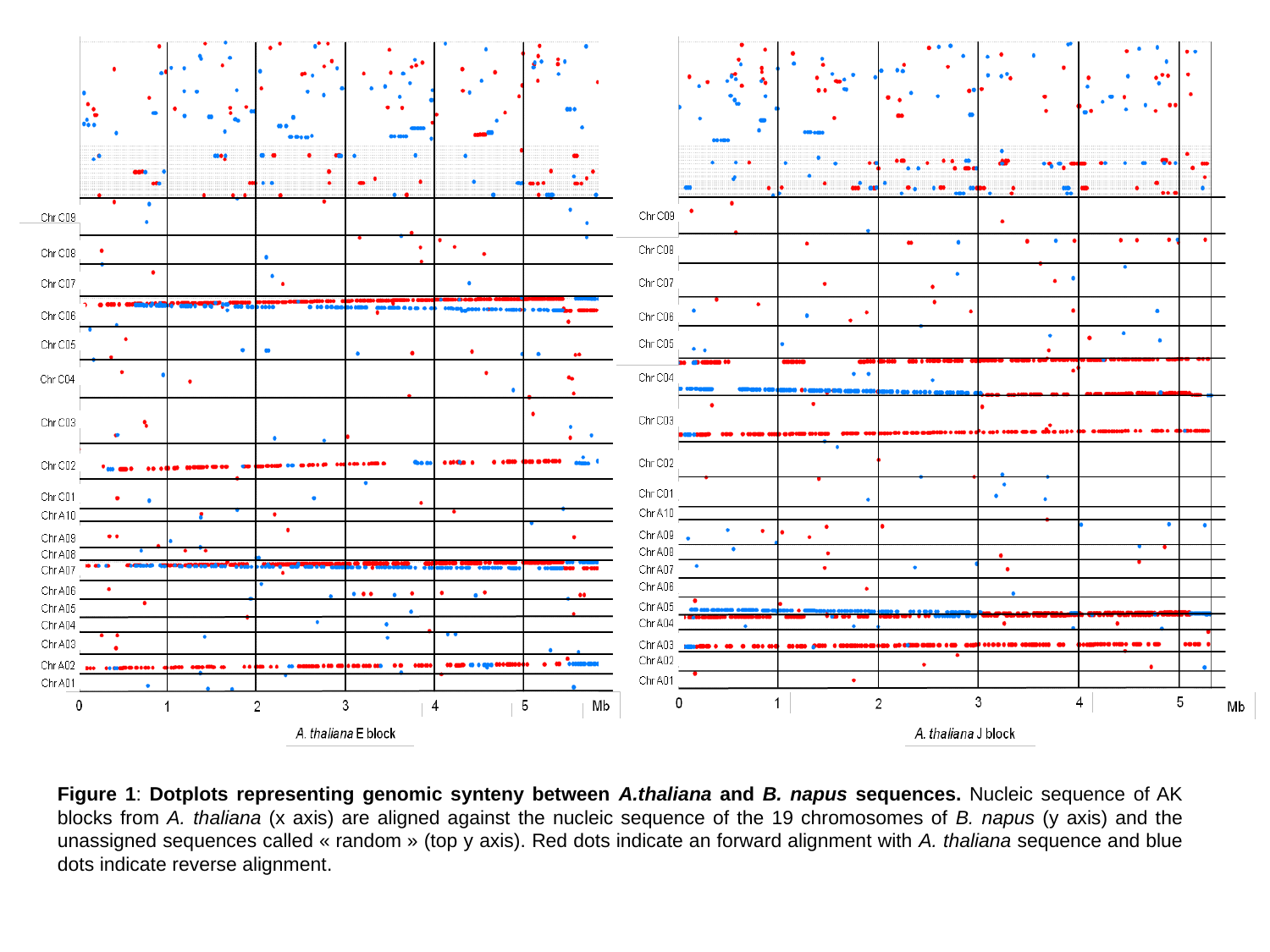

Figure 1: Dotplots representing genomic synteny between A.thaliana and B. napus sequences. Nucleic sequence of AK blocks from A. thaliana (x axis) are aligned against the nucleic sequence of the 19 chromosomes of B. napus (y axis) and the unassigned sequences called « random » (top y axis). Red dots indicate an forward alignment with A. thaliana sequence and blue dots indicate reverse alignment.

## Slide 2
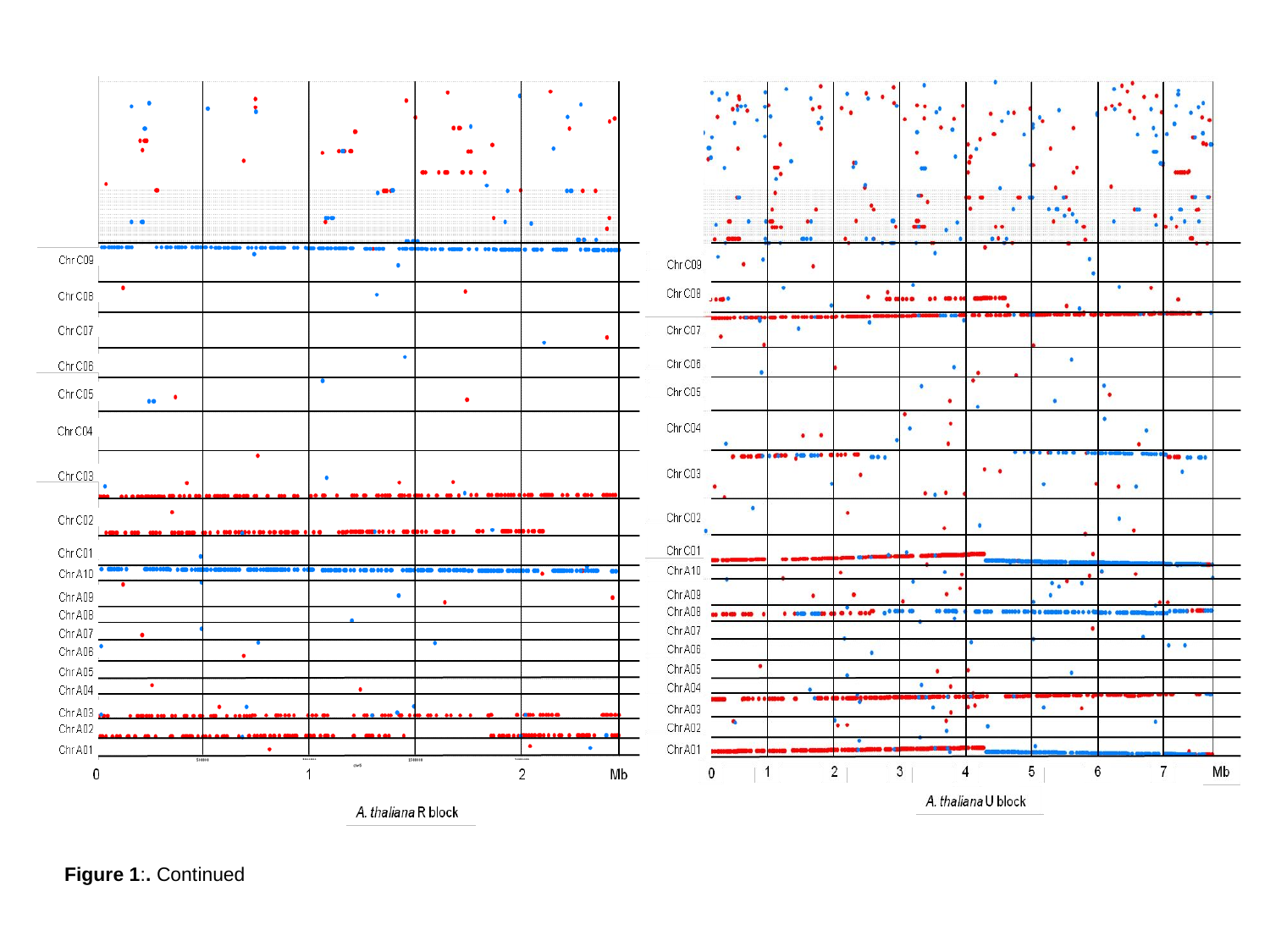

Figure 1:. Continued

## Slide 3
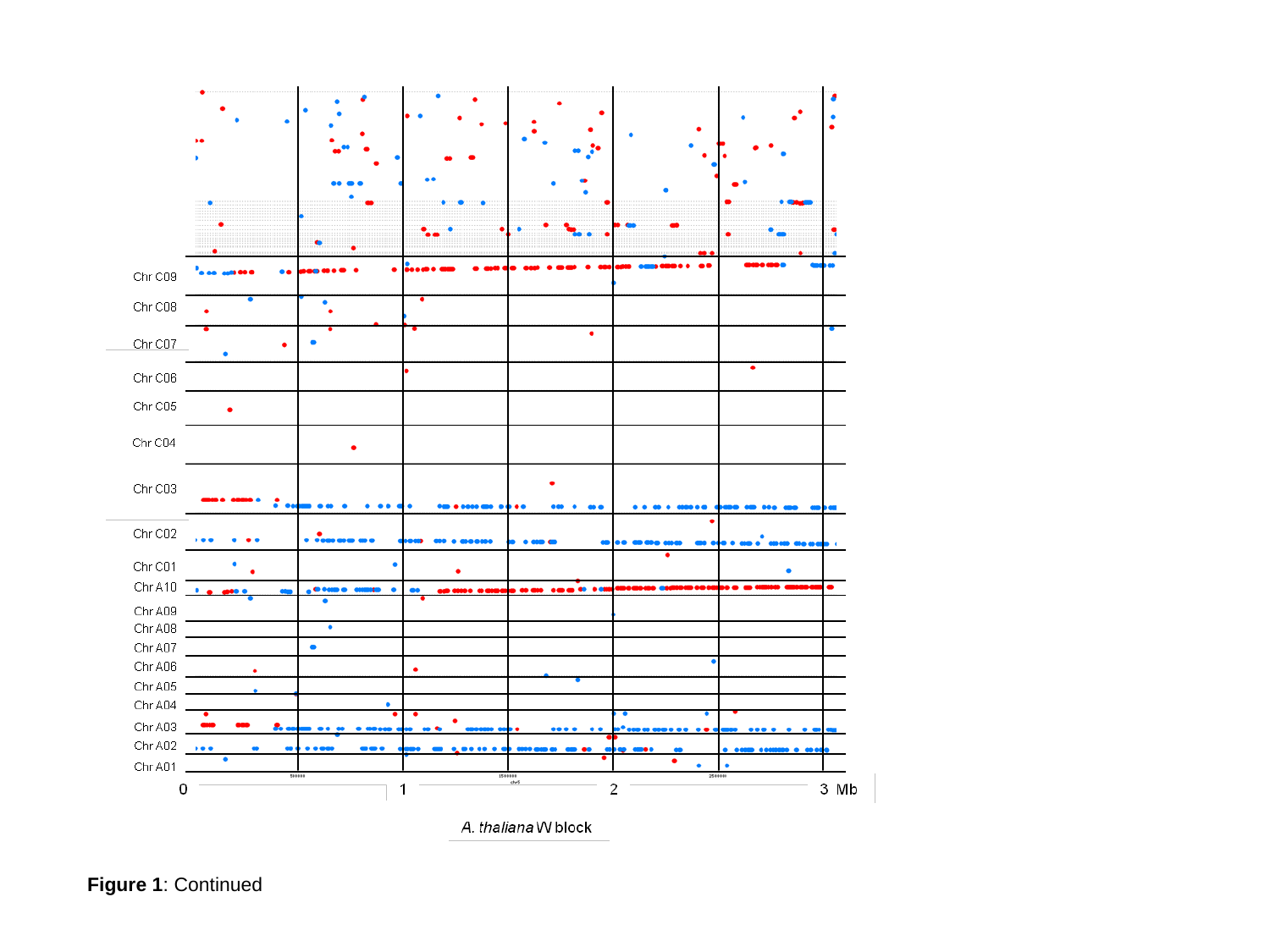

Figure 1: Continued
